# Floral Volatile Organic Compounds of Chinese *Cymbidium* Orchids: From Biosynthesis and Function to Genetic Regulation

**DOI:** 10.3390/plants15192907

**Published:** 2026-09-23

**Authors:** Zhengjing Wu, Taoran Wang, Xinrong Fu, Xun Liu, Mengting Ma, Yimiao Sun

**Affiliations:** College of Horticulture and Plant Protection, Henan University of Science and Technology, Luoyang 471000, China; wtr1887@163.com (T.W.); 15837254991@163.com (X.F.); m17637699207@163.com (X.L.); i2796448552@163.com (M.M.); ta1598458813@163.com (Y.S.)

**Keywords:** Chinese *Cymbidium* orchids, floral scent, volatile organic compounds (VOCs), terpenoids, methyl jasmonate (MeJA), terpene synthase (TPS), transcriptional regulation

## Abstract

Chinese *Cymbidium* orchids (*Cymbidium* spp.), collectively known as Guolan, are highly valued ornamental plants renowned for their elegant and persistent floral fragrance. Floral scent contributes to pollinator attraction, plant defense, and ornamental and commercial value. This review summarizes the composition of floral volatile organic compounds (VOCs), scent-emitting tissues, and spatiotemporal emission patterns in major cultivated Chinese *Cymbidium* orchids, including *C. goeringii*, *C. faberi*, *C. ensifolium*, *C. sinense*, and *C. kanran*. Floral scent emission exhibits strong tissue specificity and is dynamically regulated by floral developmental stages and circadian rhythms. Emphasis is placed on the biosynthesis and genetic regulation of the two dominant volatile classes, terpenoids and fatty acid derivatives. Current evidence indicates that terpenoids, especially sesquiterpenes, and fatty acid derivatives, particularly methyl jasmonate, are the major fragrance components, although their composition and abundance vary considerably among species. At the molecular level, terpene synthases and jasmonic acid carboxyl methyltransferase play central roles in characteristic scent formation, while multiple transcription factors regulate their expression. Future studies should clarify phenylpropanoid and benzenoid volatile biosynthesis, develop stable genetic transformation systems, and integrate multi-omics and ecological approaches to support fragrance-oriented breeding and germplasm utilization.

## 1. Introduction

Orchidaceae is one of the largest families of angiosperms and represents a highly diversified and evolutionarily successful group of monocotyledonous plants, comprising approximately 725 genera and 25,000 species across six subfamilies [1]. China harbors rich orchid diversity, with more than 170 genera and approximately 1300 species recorded [2]. Among them, Chinese *Cymbidium* orchids (commonly referred to as Guolan) represent an important terrestrial orchid group within the section Jensoa of the genus *Cymbidium*, including *Cymbidium goeringii*, *Cymbidium faberi*, *Cymbidium ensifolium*, *Cymbidium sinense*, and *Cymbidium kanran* [3,4,5,6,7] (Figure 1). These species have been cultivated for centuries and are deeply embedded in traditional Chinese horticultural culture, renowned for their elegant and characteristic floral fragrance [8].

Beyond their cultural significance, Chinese *Cymbidium* orchids exhibit high ornamental and economic value, characterized by elegant leaf morphology, diverse floral coloration, and distinctive fragrance traits [9]. However, wild populations have been increasingly threatened by over-collection and habitat degradation, highlighting the importance of germplasm conservation and sustainable utilization [10,11]. Owing to their extensive phenotypic variation and ecological adaptability, *Cymbidium* orchids also serve as valuable models for investigating the genetic and biochemical basis of plant secondary metabolism [12].

Compared with commercially dominant orchids such as *Phalaenopsis*, *Dendrobium*, and *Oncidium*, Chinese *Cymbidium* orchids are particularly valued for their delicate and persistent fragrances [13]. Nevertheless, their long growth cycles, complex genetic backgrounds, and low propagation efficiency constrain the commercial utilization and genetic improvement of elite cultivars [14]. Elucidating the biosynthesis and regulation of floral scent is therefore important for fragrance-oriented breeding and the effective use of *Cymbidium* germplasm [15].

Floral scent is primarily composed of volatile organic compounds (VOCs), which are low-molecular-weight secondary metabolites mainly including terpenoids, phenylpropanoids/benzenoids, and fatty acid derivatives [16,17]. These volatile compounds play essential ecological roles in mediating plant–pollinator interactions, enhancing resistance to biotic stresses, and facilitating adaptation to environmental conditions [18,19]. In addition, floral volatiles are widely applied in the perfume, cosmetics, food, and pharmaceutical industries, and thus represent key targets for ornamental breeding and metabolic engineering [20]. Consequently, floral scent is not only an important ornamental trait but also a powerful system for studying the evolution, biosynthesis, and regulatory networks of plant specialized metabolism.

In recent years, advances in volatile analysis techniques have significantly accelerated research on orchid floral scent [21,22]. However, due to the long life cycle, large genome size, and complex metabolic regulation in orchids, systematic understanding of floral scent biosynthesis remains incomplete, and metabolic engineering efforts are still in an early stage [23]. Current studies have revealed that orchid floral scents exhibit pronounced interspecific variation, tissue specificity, and dynamic changes across developmental stages and circadian rhythms. In Chinese *Cymbidium* orchids, numerous volatile compounds have been identified, and preliminary biosynthetic frameworks for key scent components have been proposed [24,25]. Among these, terpene synthases (TPSs), jasmonic acid carboxyl methyltransferase (JMT), and associated transcription factors are considered important candidate regulators of floral scent biosynthesis [26,27].

Despite these advances, research on floral scent in Chinese *Cymbidium* orchids remains less developed compared with traits such as flower color and morphology. Several major knowledge gaps persist. First, most studies have focused on volatile profiling and single-factor analyses, whereas a systematic understanding of how genetic background, environmental conditions, and biological interactions jointly regulate floral scent metabolism is still lacking. Second, the evolutionary and ecological significance of scent divergence among species and cultivars, as well as the coevolutionary relationships among floral scent, pollinator behavior, and human selection, remain insufficiently resolved [28,29,30,31]. Third, due to the complexity of volatile metabolic networks and extensive pathway cross-talk, current metabolic engineering strategies face significant limitations. Single-gene manipulation often fails to produce stable or predictable changes in scent profiles, partly due to metabolic redundancy, glycosylation-mediated modification, and pleiotropic effects of transcriptional regulators [32]. Moreover, existing knowledge is fragmented across species and disciplines, and a comprehensive synthesis of regulatory mechanisms underlying floral scent formation in *Cymbidium* is still lacking.

Given the increasing interest in orchid fragrance research and the growing demand for scent improvement in ornamental breeding, a systematic synthesis of floral scent diversity, biosynthetic pathways, ecological functions, and molecular regulatory mechanisms in Chinese *Cymbidium* orchids is both timely and necessary. This review summarizes current knowledge on volatile composition, emission dynamics, biosynthetic pathways, and regulatory networks in major *Cymbidium* species, with emphasis on integrating genetic, environmental, and ecological factors influencing scent formation. Finally, we highlight future research directions toward germplasm conservation, molecular breeding, and precision engineering of floral scent traits, providing a conceptual framework for advancing functional studies and applied utilization of *Cymbidium* orchids.

## 2. Overview of Floral Scent Volatile Components in Chinese *Cymbidium* Orchids

### 2.1. Detection of Floral Scent Volatile Compounds in Chinese Cymbidium Orchids

Recent advances in VOC detection technologies have greatly facilitated studies of floral scent in Chinese *Cymbidium* orchids. A relatively mature analytical workflow has been established, consisting of volatile collection and enrichment, compound separation and identification, and subsequent data analysis. Commonly used techniques include headspace solid-phase microextraction (HS-SPME), GC-MS, and dynamic headspace sampling (DHS) [33,34].

Among these methods, HS-SPME is widely applied because of its simplicity, high sensitivity, and solvent-free operation, allowing efficient in situ enrichment of floral volatiles. GC-MS remains the most frequently used analytical platform due to its excellent separation efficiency and reliable qualitative and quantitative performance. DHS is particularly suitable for long-term sampling and can more accurately reflect volatile emission dynamics under natural conditions [35,36].

Although these analytical approaches have substantially improved our understanding of floral scent composition, methodological differences can influence reported results. Variations in plant materials, sampling time, extraction temperature, extraction duration, and fiber coating type may all affect volatile profiles [37,38]. Floral scent emission in Chinese *Cymbidium* orchids often exhibits pronounced circadian fluctuations, resulting in significant differences in volatile composition and relative abundance among samples collected at different time points [39]. Furthermore, elevated extraction temperatures or prolonged extraction periods may alter the proportions of thermolabile terpenoids and potentially induce degradation of certain volatile constituents, thereby reducing comparability among studies [40].

These analytical approaches have provided essential information for characterizing floral scent diversity and comparing volatile profiles among Chinese *Cymbidium* species (Table 1).

### 2.2. Major Classes of Floral Scent Volatiles in Chinese Cymbidium Orchids

Floral scent volatiles in Chinese *Cymbidium* orchids can be broadly classified into three major groups: terpenoids, phenylpropanoids/benzenoids, and fatty acid derivatives [60]. Terpenoids generally represent the most abundant and diverse class and are primarily responsible for the characteristic fragrance of Chinese *Cymbidium* orchids. Fatty acid derivatives, particularly methyl jasmonate and its related compounds, predominate in certain species, whereas phenylpropanoids and benzenoids, despite their lower abundance, make important contributions to fragrance quality and complexity [41,61,62]. Variation in the composition and relative abundance of these compounds collectively generates the diverse volatile composition observed among different Chinese *Cymbidium* species (Table 2).

#### 2.2.1. Floral Scent Composition of Chinese *Cymbidium* Orchids

Terpenoids represent the predominant floral volatiles in *C. goeringii*, with sesquiterpenes accounting for the largest proportion. Nerolidol is consistently detected as one of the most abundant compounds and is regarded as a key contributor to the characteristic fragrance of this species. Other commonly identified sesquiterpenes include farnesene, caryophyllene, and α-selinene. In addition, phenylpropanoid and benzenoid compounds such as phenylethanol and benzyl alcohol, together with fatty acid derivatives including methyl jasmonate and nonanal, contribute to the overall scent profile.

Floral scent in *C. faberi* is characterized by a complex mixture of terpenoids, jasmonates, and alcohols. MeJA and epi-methyl jasmonate (epi-MeJA) are consistently identified as dominant volatile constituents across multiple populations. Additional important compounds include farnesol, ocimene, and nerolidol.

MeJA and epi-MeJA constitute the major volatile compounds in *C. ensifolium* and are considered key contributors to its characteristic floral aroma. Their abundance changes dynamically during flower development and typically reaches a maximum at full anthesis. Terpenoid components are mainly represented by sesquiterpenes such as farnesol, β-farnesene, and β-bisabolene, which contribute substantially to the overall scent profile.

The floral scent of *C. sinense* is dominated by terpenoids and fatty acid derivatives, with terpenoids representing the major volatile fraction. Important monoterpenes include linalool, α-terpineol, α-pinene, and limonene, whereas major sesquiterpenes include farnesol, β-ionone, and α-farnesene. Compared with other Chinese *Cymbidium* species, *C. sinense* generally contains a higher proportion of monoterpenes, resulting in a distinctive volatile profile.

Sesquiterpenes and fatty acid derivatives constitute the major volatile classes in *C. kanran*. Representative compounds include MeJA, epi-MeJA, farnesol, farnesyl acetate, and α-bergamotene. Considerable variation in volatile composition has been observed among geographical populations. Some populations are characterized by relatively high levels of fruity and green-note volatiles, whereas others contain larger proportions of floral and fatty-acid-derived compounds (Table 2).

#### 2.2.2. Comparative Analysis of Floral Scent Composition Among Major Chinese *Cymbidium* Orchids

Although terpenoids and fatty acid derivatives dominate the floral scent profiles of *C. goeringii*, *C. faberi*, *C. ensifolium*, *C. sinense*, and *C. kanran*, marked interspecific differences are evident. *C. goeringii* is characterized by sesquiterpenes such as nerolidol, whereas *C. faberi* and *C. ensifolium* contain high proportions of MeJA. In contrast, *C. sinense* is enriched in monoterpenes, including linalool, together with characteristic compounds such as β-ionone. The scent profile of *C. kanran* is primarily determined by the combined contribution of MeJA and sesquiterpenes. These interspecific differences likely reflect the combined effects of genetic background, environmental adaptation, and long-term artificial selection.

#### 2.2.3. Factors Influencing Variations in Floral Volatile Profiles of Chinese *Cymbidium* Orchids

Although numerous volatile compounds have been identified in Chinese *Cymbidium* orchids, considerable variation exists among reported floral scent profiles, even within the same species. Such discrepancies likely result from the combined effects of genetic background, environmental conditions, circadian regulation, sampling strategies, and analytical methodologies [63].

Geographic and environmental factors, including temperature, light intensity, precipitation, and cultivation conditions, can influence floral scent composition by altering plant physiological status, metabolic activity, and volatile emission patterns [12]. Table 1 illustrates this variation in *C. goeringii*: studies conducted in Beijing, Guizhou, the Qinling Mountains, Taiwan, and Jiangxi commonly reported nerolidol and/or farnesol among the major volatiles [43,45,46,47,48,49,50], whereas α-selinene, dodecane, *n*-tetradecane, and thujopsene were reported in Zhejiang [44], and α-caryophyllene, *p*-methylanisole, benzyl alcohol, and phenylethyl alcohol in Sichuan [51]. In addition, floral scent release is often regulated by circadian rhythms, with many compounds exhibiting time-dependent emission patterns associated with specific developmental stages and potential pollinator activity [64,65]. Differences in sampling time and developmental stage may therefore substantially affect the reported volatile profiles.

Methodological variation also contributes to discrepancies among studies. Differences in volatile collection procedures, extraction conditions, and analytical parameters may influence the detection and quantification of specific compounds, particularly unstable or low-abundance volatiles [12]. Even among headspace solid-phase microextraction coupled with gas chromatography–mass spectrometry (HS-SPME-GC–MS) studies of *C. goeringii*, extraction temperatures ranged from 20 to 80 °C, while extraction times ranged from 5 to 90 min [43,44,47,50]. Such differences may substantially affect extraction efficiency and the relative abundance of detected compounds. Moreover, species-specific metabolic characteristics and differential regulation of biosynthetic pathways further contribute to interspecific and population-level variation in volatile composition [66].

Accordingly, comparisons among studies should consider the combined effects of genetic, environmental, temporal, and methodological factors. Because geographic origin and analytical conditions often differ simultaneously among studies, the observed variation should not be attributed solely to regional or genetic differences. Standardized sampling and analytical protocols, combined with integrative multi-omics approaches, will be essential for accurately characterizing floral scent diversity and its regulatory mechanisms in Chinese *Cymbidium* orchids (Table 1).

### 2.3. Floral Scent Emission Sites and Tissue Specificity in Chinese Cymbidium Orchids

The distribution of floral scent volatiles among floral organs exhibits pronounced tissue specificity in Chinese *Cymbidium* orchids. Comparative studies have demonstrated that different species employ distinct floral organs as major sites of volatile production and emission (the structural diagram of the floral organs of Chinese orchids is shown in Supplementary Figure S1), indicating substantial interspecific variation in scent emission strategies.

In some commercially cultivated orchids, scent emission is concentrated in a specific floral organ. For example, the labellum is the principal scent-emitting organ in fragrant *Phalaenopsis* ‘Chanel’ [67], whereas the ‘sepals’ or labellum predominate in certain *Dendrobium* species [68]. By contrast, scent emission in Chinese *Cymbidium* orchids generally involves multiple floral organs, whose relative contributions vary among species. Their complex scent bouquets comprise diverse volatile classes, particularly sesquiterpenes, MeJA-related compounds, and other fatty acid derivatives.

Among the species investigated, *Cymbidium faberi* exhibits the most distinct functional differentiation among floral organs. The column contains the greatest number of detected volatiles, mainly alcohols and esters. ’Sepals’ and ‘petals’ mainly emit terpenoid compounds such as pinene and ocimene, whereas ‘petals’ accumulate relatively high levels of farnesol and MeJA. In contrast, the labellum contains only a limited number of volatile constituents and contributes minimally to total scent emission [26,69]. A different emission pattern has been reported in *C. ensifolium*. ‘Petals’ exhibit the highest volatile emission rates and are dominated by MeJA and related isomers. ‘Sepals’ possess the greatest chemical diversity and serve as the principal source of sesquiterpenes. The column contributes little to volatile release, while the labellum emits only trace amounts of floral volatiles [55]. In *C. sinense*, all floral organs contribute to scent emission, although substantial differences occur in both volatile composition and emission intensity. ‘Sepals’ exhibit the highest total volatile abundance, whereas ‘petals’ contain the greatest diversity of volatile compounds. The column is enriched in aldehydes such as hexanal, producing a distinct volatile profile, while the labellum displays relatively low volatile diversity and abundance [58].

Overall, the sepals and petals are the predominant scent-emitting organs in most Chinese *Cymbidium* orchids examined, whereas the labellum generally makes a limited contribution. However, the relative importance of individual organs is species-dependent: *C. faberi* relies mainly on the column and ‘petals’, *C. ensifolium* on the ‘petals’ and ‘sepals’, and *C. sinense* on a more distributed emission system. This tissue-specific differentiation may reflect adaptation to distinct pollination systems and evolutionary histories.

### 2.4. Developmental Regulation of Floral Scent Emission in Chinese Cymbidium Orchids

Floral scent emission is developmentally regulated in orchids. In *Phalaenopsis* and *Dendrobium*, emission generally increases during flower opening, peaks at anthesis, and declines during senescence, with linalool and other terpenoids as major contributors [67,68]. Chinese *Cymbidium* orchids exhibit more diverse developmental patterns involving changes in both emission intensity and the relative abundance of terpenoids, fatty acid derivatives, and aromatic compounds.

In *C. goeringii*, floral scent composition shifts from hydrocarbon- and ester-rich profiles at the bud stage to terpenoid-dominated profiles during anthesis. Volatiles associated with green and fruity odors are abundant in unopened flowers, whereas compounds such as nerolidol, geranylacetone, farnesol, and β-farnesene accumulate rapidly after flower opening. Farnesol emission typically reaches its maximum on the second day after anthesis [47,50]. The floral scent of *C. faberi* exhibits a clear developmental transition. Aromatic compounds dominate during the bud stage, whereas MeJA and its isomers become abundant during partial flower opening. At full bloom, terpenoids including ocimene and farnesol constitute the major volatile fraction. During floral senescence, aldehydes and long-chain alkanes gradually increase in relative abundance [42]. A distinct developmental pattern is observed in *C. ensifolium*. Floral volatiles are scarce during the bud stage, with eucalyptol representing one of the few detectable compounds. MeJA and related isomers accumulate progressively during flower opening and reach their highest levels at full bloom, typically peaking on the fourth day after anthesis. Thereafter, total volatile emission declines markedly during senescence [48].

Overall, floral scent emission in Chinese *Cymbidium* orchids displays pronounced developmental regulation. However, species differ substantially in the dominant volatile classes, timing of maximum emission, and developmental trajectories of scent production. These differences likely reflect species-specific strategies for resource allocation and reproductive adaptation.

### 2.5. Diurnal and Circadian Regulation of Floral Scent Emission in Chinese Cymbidium Orchids

Diurnal regulation of floral scent emission is widespread in orchids. In *Phalaenopsis* and *Dendrobium*, emission commonly peaks during the day, with monoterpenes representing major rhythmic components [31,67,68]. Chinese *Cymbidium* orchids exhibit species-specific changes in both total emission and volatile composition throughout the day, particularly in terpenoids, MeJA-related compounds, and aldehydes.

In *C. faberi*, floral scent composition changes substantially throughout the day. Terpenoids such as ocimene and various esters showed different emission patterns at different daytime sampling points, whereas the relative abundance of farnesol and MeJA increases during the afternoon, indicating temporal shifts in volatile composition [42]. Compared with *C. faberi*, *C. kanran* exhibits relatively weak diurnal variation. Aldehydes such as hexanal remain abundant throughout the day, and only minor fluctuations are observed in low-abundance volatile compounds, resulting in a comparatively stable scent profile [59]. The strongest circadian regulation has been reported in *C. ensifolium*. Peak volatile emission generally occurs around midday, with MeJA and its derivatives representing the dominant daytime compounds. During the night, aldehydes become increasingly abundant and gradually dominate the volatile profile [48].

Further studies have demonstrated that MeJA emission is regulated by both environmental light signals and the endogenous circadian clock. Under normal photoperiod conditions, emission exhibits a stable rhythmic pattern. Continuous light reduces rhythmic amplitude and extends the oscillation period, whereas continuous darkness markedly suppresses MeJA emission and abolishes rhythmicity [70]. These observations suggest that floral scent emission in Chinese *Cymbidium* orchids is controlled through interactions between external environmental cues and endogenous timing mechanisms.

Overall, Chinese *Cymbidium* orchids exhibit distinct species-specific temporal emission patterns. These rhythms may synchronize floral scent release with pollinator activity while minimizing metabolic costs, thereby contributing to pollination efficiency and reproductive success.

### 2.6. Ecological Functions of Floral Volatiles and Floral Architecture in Pollination of Chinese Cymbidium Orchids

Floral volatile compounds are long-distance chemical signals mediating plant–pollinator interactions and represent key ecological traits shaped during orchid evolution. Unlike many tropical orchids that rely on conspicuous floral colors for pollinator attraction, Chinese *Cymbidium* orchids generally possess relatively small flowers with subtle coloration, resulting in weak visual signals in natural habitats [71]. However, these orchids release species-specific volatile blends that enhance flower recognition and localization by pollinators, compensating for their limited visual attractiveness [72]. Therefore, floral scent may represent a particularly important signal for pollinator attraction in maintaining pollination interactions of Chinese *Cymbidium* orchids.

Scent emission in Chinese *Cymbidium* orchids is closely associated with floral organ differentiation, and different organs exhibit functional specialization during pollination. ‘Sepals’, ‘petals’, and the column are generally the major scent-emitting tissues, whereas the labellum primarily contributes to pollinator guidance and pollen transfer, although its contribution to scent emission varies among species [73]. In *Cymbidium ensifolium*, ‘petals’, ‘sepals’, and the column released abundant VOCs, including sesquiterpenes, esters, ketones, phenolic compounds, hydrocarbons, and unsaturated fatty acid derivatives, while the labellum showed lower volatile diversity and emission levels [73]. Ultrastructural observations revealed that scent-producing tissues contained abundant plastids, developed endoplasmic reticulum (ER), osmiophilic droplets, and intact mitochondria, suggesting their involvement in volatile biosynthesis, storage, and transport [74]. Plastids may serve as important sites for terpenoid synthesis, while ER and osmiophilic droplets may contribute to the formation, transport, and storage of lipid-derived volatiles [75]. By contrast, the labellum contains fewer plastids, less-developed ER, and fewer osmiophilic droplets, supporting its predominantly mechanical and guiding functions.

Specialized floral architecture acts together with scent to enhance pollination specificity. The labellum provides a landing platform and, together with the petals, lateral lobes, and column, forms a restricted pathway that directs pollinator movement and pollen transfer [76]. In *Cymbidium goeringii*, the Chinese honeybee (*Apis cerana cerana*) is attracted by floral scent and lands on the labellum. During flower entry and exit, the bee uses labellar structures for support, and its thorax contacts the viscidium of the column, facilitating pollen removal and deposition. In contrast, larger bees such as *Anthophora melanognatha* may respond to floral scent but fail to achieve effective pollination because their body size prevents successful entry into the floral chamber [77]. These findings indicate that floral architecture acts together with chemical signals to filter potential pollinators and enhance pollination specificity.

The floral scents of Chinese *Cymbidium* orchids consist mainly of terpenoids, alcohols, esters, aldehydes, ketones, and hydrocarbons. Several odor-active compounds affect insect olfactory and behavioral responses. For example, α-pinene is an important component of the floral scent of *C. goeringii* and may contribute to honeybee attraction [54]. Recent studies showed that α-pinene induced strong attraction responses in honeybees, even exceeding the response to benzaldehyde, suggesting that monoterpenes may serve as important chemical cues in orchid–pollinator communication. In addition, 2-methylbutanal was identified as a shared odor-active compound in *C. faberi* and *C. sinense* and elicited strong antennal electrophysiological responses in honeybees [78,79]. However, individual compounds alone cannot fully explain pollinator attraction, indicating that orchid scent recognition depends on complex volatile blends involving compound composition, relative abundance, concentration, and synergistic interactions.

Most Chinese *Cymbidium* species are rewardless and provide little or no nectar or other nutritional resources. They therefore depend on the integration of chemical attraction and precise floral architecture for effective pollination. In *C. goeringii* and *C. faberi*, floral scent attracts potential pollinators, whereas the labellum, column, and pollinarium control visitor positioning and pollen transfer [54,72,77,79,80]. This coordinated system enables successful reproduction without direct floral rewards.

Despite this specialization, natural reproductive success is generally low because most species depend on external pollinators. In *C. goeringii*, pollen removal reached approximately 65.79%, whereas natural fruit set was only 6.67%; bagged flowers failed to reproduce, confirming pollinator dependence. Artificial self- and cross-pollination produced high fruit set, indicating that reproduction is limited primarily by insufficient pollinator visitation rather than incompatibility. Similarly, wild *C. faberi* exhibited a natural fruit set of approximately 7.5%, compared with nearly 100% following artificial pollination [77,79]. Pollinator abundance and behavior, together with the attractiveness of floral scent, therefore strongly constrain reproductive success.

Overall, pollination in Chinese *Cymbidium* orchids depends on the integration of species-specific volatile signals and specialized floral architecture. Floral scent attracts and guides potential pollinators, whereas floral structures filter visitors and ensure precise pollen transfer. This coordination may promote pollinator specialization, reproductive isolation, and diversification within the genus, highlighting the importance of floral VOCs in the ecological success and evolution of rewardless orchids (Figure 2).

## 3. Biosynthesis and Regulatory Mechanisms of Floral Scent Compounds in Chinese *Cymbidium* Orchids

Floral scent in Chinese *Cymbidium* orchids is primarily composed of terpenoids, fatty acid derivatives, and phenylpropanoid/benzenoid compounds [61,81]. Among these volatile classes, the biosynthetic mechanisms of terpenoids and methyl jasmonate-derived compounds have received the greatest research attention, and several key structural genes have been identified and functionally characterized. In contrast, the biosynthetic pathways and regulatory mechanisms underlying phenylpropanoid and benzenoid volatiles remain poorly understood, and the corresponding enzymes and regulatory factors have yet to be systematically characterized. Consequently, integrating genomics, transcriptomics, metabolomics, and functional genomics will be essential for constructing comprehensive regulatory networks governing floral scent formation in Chinese *Cymbidium* orchids.

### 3.1. Terpenoid Biosynthesis and the TPS Gene Family in Chinese Cymbidium Orchids

Terpenoids constitute the largest and most diverse class of floral scent volatiles in Chinese *Cymbidium* orchids. Their biosynthesis is derived from two interconnected metabolic pathways: the cytosolic mevalonate (MVA) pathway and the plastidial methylerythritol phosphate (MEP) pathway [81,82,83] (Figure 3).

Although both pathways provide the universal isoprenoid precursors required for terpene biosynthesis, the final composition of floral terpenoids is largely determined by TPS. TPS enzymes catalyze the conversion of prenyl diphosphate substrates into structurally diverse terpene compounds and therefore represent key regulatory nodes controlling floral scent composition [84,85,86].

Phylogenetic analyses indicate that TPS genes identified in Chinese *Cymbidium* orchids are mainly distributed among the TPS-a, TPS-b, TPS-c, and TPS-e/f subfamilies. Members of the TPS-a subfamily are primarily associated with sesquiterpene formation, whereas TPS-b genes predominantly participate in monoterpene biosynthesis. TPS-e/f members are mainly involved in diterpene metabolism. Expression analyses further reveal pronounced tissue specificity and developmental regulation of TPS genes, with transcript abundance often showing strong correlations with the emission patterns of corresponding terpene volatiles [87].

Comparative genomic studies have revealed substantial variation in TPS gene family size among *Cymbidium* species. In *Cymbidium goeringii*, 40 TPS genes have been identified, and functional characterization demonstrated that CgTPS7 participates in sesquiterpene biosynthesis and contributes to floral scent formation [47,88]. In *C. sinense*, the TPS family has expanded to 59 members spanning five subfamilies, including TPS-a, TPS-b, TPS-c, TPS-d, and TPS-e/f. Notably, expansion of the TPS-b subfamily has generated multiple α-terpineol synthase candidates, several of which are highly expressed during anthesis and are likely associated with the characteristic fragrance of this species [57].

In *C. ensifolium*, several terpene biosynthetic genes, including CeGDPS, CeFDPS, and CeLIS, have been identified. Among them, CeLIS belongs to the TPS-b subfamily and directly catalyzes the conversion of GPP into linalool. Its expression pattern closely parallels linalool emission during flower development [89]. Similarly, 32 TPS genes have been identified in *C. faberi*, including 13 TPS-a, 15 TPS-b, and four TPS-e/f members. Functional analysis demonstrated that CfTPS18 catalyzes the production of multiple monoterpenes, including β-myrcene, geraniol, and α-pinene, and exhibits high expression levels in fully opened flowers [90].

Additional evidence supporting TPS functional diversification has been obtained from studies of *Cymbidium* hookerianum. Functional characterization revealed that CtTPS1 primarily produces linalool from GPP, whereas CtTPS2, CtTPS3, and CtTPS8 utilize FPP to generate sesquiterpenes such as (E)-β-caryophyllene, valencene, and α-humulene, respectively [91]. These findings highlight the extensive biochemical diversification of TPS enzymes within the genus *Cymbidium* and suggest that TPS evolution has played a central role in the diversification of floral scent profiles.

Overall, expansion and functional divergence of the TPS gene family appear to be major drivers of floral scent diversification in Chinese *Cymbidium* orchids. Current studies have largely focused on gene identification and enzymatic characterization, whereas the upstream regulatory networks governing TPS expression remain poorly understood. Future research should combine multi-omics approaches, transcriptional regulatory analyses, and genetic transformation technologies to elucidate the molecular networks controlling terpene biosynthesis and floral scent formation in Chinese *Cymbidium* orchids.

### 3.2. Biosynthesis and Regulation of Methyl Jasmonate in Chinese Cymbidium Orchids

MeJA is the volatile methyl ester of jasmonic acid (JA) and represents the only volatile compound within the jasmonate family [92]. In Chinese *Cymbidium* orchids, MeJA is widely distributed among species such as *Cymbidium faberi*, *C. ensifolium,* and *C. kanran*, where it constitutes one of the major contributors to floral scent. In addition to its role in fragrance formation, MeJA functions as an important signaling molecule involved in plant growth, development, stress responses, and defense regulation [93,94,95] (Figure 3).

#### 3.2.1. Key Structural Genes Involved in MeJA Biosynthesis

Studies of MeJA biosynthesis in Chinese *Cymbidium* orchids have primarily focused on genes encoding enzymes of the α-linolenic acid pathway, including LOX, AOS, AOC, OPR, and JMT. Among these genes, JMT has attracted particular attention because it catalyzes the terminal step leading directly to volatile MeJA production.

In *C. faberi*, CfJMT was isolated from fully opened flowers and was shown to encode a typical member of the SABATH methyltransferase family. Expression analyses revealed strong enrichment of CfJMT transcripts in floral tissues, including petals, sepals, and labella, where transcript abundance closely paralleled MeJA accumulation patterns [95]. Furthermore, upstream pathway genes such as CfLOX3, CfAOS, and CfAOC are also highly expressed during anthesis, indicating coordinated activation of the entire α-linolenic acid pathway during floral scent formation [26,96].

In *C. ensifolium*, transcriptomic and enzymatic analyses identified several candidate genes involved in MeJA biosynthesis, including CeLOX, CeAOS, CeAOC, CeOPR3, and CeJMT [55,94]. Functional assays demonstrated that CeJMT is capable of catalyzing the conversion of JA into MeJA in vitro. These genes exhibit pronounced tissue specificity and developmental regulation, with transcript abundance increasing during flower development and reaching maximum levels during the early flowering stage. In addition, their expression patterns display strong circadian rhythmicity and closely correspond to temporal changes in MeJA emission [97].

Although direct functional evidence remains limited in *C. goeringii*, transcriptome analyses have identified multiple jasmonate-related genes whose expression profiles suggest active involvement of the MeJA pathway in floral scent formation [47].

Collectively, current evidence supports a conserved biosynthetic framework in Chinese *Cymbidium* orchids, extending from the LOX–AOS–AOC module to the terminal JMT-catalyzed methylation step. Among these enzymes, JMT appears to represent the most critical regulatory node governing volatile MeJA production.

#### 3.2.2. Functional Characterization of JMT Genes and Current Challenges

Despite considerable progress in gene identification, functional validation of JMT genes remains limited. Most available evidence is derived from heterologous expression experiments involving CfJMT from *C. faberi*.

Transgenic expression of CfJMT in petunia failed to significantly increase MeJA accumulation or alter floral scent composition, although changes in leaf morphology and pollen development were observed [95]. Similarly, transient expression in *Phalaenopsis* accelerated petal senescence and color fading without producing detectable increases in MeJA content [96]. Overexpression of CfJMT in tomato also failed to substantially enhance MeJA levels but resulted in stronger wound-induced jasmonate signaling responses [98,99]. Compared with CfJMT, CeJMT has only been functionally characterized through in vitro enzyme assays, and evidence from stable genetic transformation is currently unavailable [94]. These findings collectively suggest that MeJA biosynthesis is not controlled solely by JMT activity. Instead, substrate availability, metabolic flux allocation, precursor transport, and transcriptional regulation are likely to jointly determine final MeJA accumulation levels.

Therefore, establishing efficient and stable transformation systems for Chinese *Cymbidium* orchids remains a major prerequisite for elucidating the in vivo functions of JMT and other jasmonate-related genes. Functional studies conducted in homologous genetic backgrounds will be essential for accurately assessing their contributions to floral scent formation.

#### 3.2.3. Transcriptional Regulation of MeJA Biosynthesis

Recent studies have demonstrated that MeJA biosynthesis is subject to complex transcriptional regulation. In *C. faberi*, promoter analyses revealed that CfAOC exhibits constitutive promoter activity, whereas the CfJMT promoter displays strong floral tissue specificity and contains multiple cis-regulatory elements, including G-box motifs [98].

Several transcription factors have been identified as potential regulators of the MeJA pathway. Functional analyses demonstrated that Cfb*HLH*5 activates CfAOC promoter activity, whereas Cfb*HLH*36 represses CfJMT transcription, indicating that members of the b*HLH* family may function as both positive and negative regulators of jasmonate biosynthesis [100]. In addition, multiple MYB transcription factors significantly activate the promoters of both CfAOC and CfJMT, and several of these genes exhibit elevated expression during anthesis [101].

Transcriptome analyses further revealed significant enrichment of *MYB*- and b*HLH*-related regulatory pathways during floral scent formation in *C. faberi*. These transcription factors may collectively regulate MeJA metabolism through mechanisms analogous to the *MYC*/JAM regulatory module described in other plant species [99].

In contrast, transcriptional regulation of MeJA biosynthesis remains largely unexplored in *C. ensifolium* and *C. goeringii*. Although several candidate regulators, including b*HLH*13, *WRKY*33, and *VIP*1, have been identified through transcriptomic analyses, direct evidence linking these factors to regulation of the α-linolenic acid pathway is still lacking [102].

Overall, current knowledge of the transcriptional regulatory network governing MeJA biosynthesis in Chinese *Cymbidium* orchids remains fragmentary. Available evidence indicates that MYB and b*HLH* transcription factors play central regulatory roles; however, the hierarchical relationships among these regulators, their downstream targets, and their interactions with environmental signals remain poorly understood. Future studies integrating gene editing, chromatin-level analyses, and multi-omics approaches will be necessary to establish a comprehensive regulatory framework for MeJA-mediated floral scent formation.

## 4. Factors Influencing Floral Scent Formation in Chinese *Cymbidium* Orchids

Floral scent formation in Chinese *Cymbidium* orchids is a complex trait jointly regulated by genetic background, metabolic networks, environmental cues, developmental programs, and multilayered molecular signaling pathways. These factors collectively determine the composition, abundance, and emission dynamics of VOCs by modulating their biosynthesis, transport, storage, and release (Figure 4).

### 4.1. Genetic and Metabolic Regulation

Genetic background and metabolic capacity constitute the primary determinants of floral scent composition. Among the biosynthetic enzymes involved in volatile formation, terpene synthases play a central role in controlling the diversity and structural complexity of terpenoid volatiles [103,104]. Expansion and functional diversification of the TPS gene family are therefore key contributors to floral scent variation in Orchidaceae [87].

Beyond TPS catalytic activity, upstream precursor supply and metabolic flux allocation also play critical roles in determining floral scent composition in orchids. The MEP pathway provides GPP for monoterpene biosynthesis, whereas the MVA pathway supplies FPP for sesquiterpene formation. Competition for shared precursors, including IPP and DMAPP, and differential carbon flux distribution between these pathways contribute to species-specific floral scent chemotypes [82].

A notable feature of TPS enzymes is their catalytic promiscuity, whereby a single enzyme can utilize one or multiple prenyl diphosphate substrates to generate structurally diverse volatile products. This functional flexibility is closely associated with variations in active site architecture and key amino acid residues [105]. For example, CfTPS18 from *Cymbidium faberi* catalyzes the formation of multiple monoterpenes, including β-myrcene, geraniol, and α-pinene [90]. Similarly, PbTPS3 from *Phalaenopsis* exhibits dual linalool and cis-ocimene synthase activity [106], whereas DcTPSb1 from *Dendrobium chrysotoxum* produces a range of monoterpenes and sesquiterpenes, including linalool, ocimene, and α-terpineol [107]. These findings highlight TPS multifunctionality as a key biochemical basis for floral scent diversity in Orchidaceae.

In addition to enzymatic specificity, intracellular metabolic environments also influence volatile formation [108]. Variations in vacuolar pH can alter the stability and isomerization of monoterpene alcohols, thereby affecting final scent composition [109]. Acidic conditions may promote rearrangement reactions, whereas neutral conditions generally favor volatile stability and accumulation [110]. Thus, subcellular physicochemical conditions represent an additional layer of regulation in floral scent biosynthesis.

Furthermore, precursor availability is a critical limiting factor in volatile production [111]. Floral scent biosynthesis depends not only on the activity of terminal synthases but also on upstream metabolic flux distribution and substrate supply [19,112]. When multiple pathways compete for shared precursors, substrate allocation becomes a key determinant of final volatile profiles. Consequently, effective metabolic engineering of floral scent traits requires coordinated optimization of both precursor supply and downstream enzymatic steps rather than manipulation of single structural genes alone.

Following biosynthesis, volatile compounds must undergo intracellular storage, transport, and diffusion before emission. Therefore, floral scent output is determined not only by biosynthetic capacity but also by cellular compartmentation, membrane permeability, and physicochemical properties of volatile molecules.

### 4.2. Environmental and Ecological Regulation

Environmental factors exert strong regulatory effects on floral scent formation and emission in Chinese *Cymbidium* orchids, with light and temperature representing the most influential variables [113].

Light functions both as an energy source and as a signaling cue regulating secondary metabolism. As understory plants, Chinese *Cymbidium* orchids typically exhibit enhanced volatile accumulation under moderate shading conditions, whereas excessive or insufficient light can suppress scent biosynthesis [8]. In addition to light intensity, light quality plays a regulatory role in volatile emission. In *Cymbidium ensifolium*, continuous darkness significantly reduces methyl jasmonate emission, whereas blue light can partially restore emission levels, suggesting a major role for blue-light signaling pathways in regulating scent biosynthesis. In contrast, red light shows limited effects [66,67,70].

Temperature regulates floral scent primarily through its effects on enzyme activity, metabolic flux, and volatile evaporation rates. Moderate temperature increases generally enhance volatile biosynthesis and emission, whereas low temperatures reduce metabolic activity and weaken scent intensity. High humidity, adequate aeration, and suitable substrate pH further support normal floral organ development and volatile accumulation [8,112].

From an ecological perspective, environmental regulation of floral scent contributes to habitat adaptation and improves pollinator attraction efficiency, suggesting that scent variation is closely linked to ecological niche specialization in Chinese *Cymbidium* orchids.

### 4.3. Developmental and Circadian Regulation

Floral scent emission is highly dynamic and is tightly coordinated with floral developmental processes and endogenous timing systems.

During flower development, volatile composition and emission intensity undergo continuous reprogramming, typically reaching maximum levels at anthesis, followed by a rapid decline during senescence. This pattern reflects a resource allocation strategy optimized for reproductive success, in which scent emission is prioritized during the pollination window and reduced after fertilization [112].

In addition to developmental regulation, floral scent emission exhibits pronounced circadian rhythmicity. In *C. ensifolium*, methyl jasmonate emission follows a stable diurnal oscillation that aligns with peak pollinator activity [70]. This rhythm is jointly regulated by photoperiodic signals and the endogenous circadian clock. Continuous darkness disrupts rhythmicity, indicating that light serves as an essential entraining signal (zeitgeber) for temporal regulation of volatile emission.

Spatial regulation further contributes to scent specificity. Genes involved in terpene biosynthesis are preferentially expressed in floral tissues such as ‘petals’ and ‘sepals’, which correspond to primary emission sites. This spatial coordination between gene expression and volatile release ensures efficient ecological function and optimized resource utilization [114].

### 4.4. Molecular Regulation and Signal Transduction Networks

Floral scent biosynthesis is governed by multilayered transcriptional and post-transcriptional regulatory networks that integrate environmental signals with endogenous developmental programs.

Multiple transcription factor families, including *AP2/ERF*, *MYB*, *bHLH*, *bZIP*, and *WRKY*, have been implicated in the regulation of volatile biosynthesis pathways [96]. In *C. ensifolium*, the transcription factor Ce*MYC*2 directly binds to the promoter of CeAOC, a key gene in the jasmonate biosynthetic pathway, and interacts with the light-signaling component CeCOP1. This regulatory module integrates jasmonate signaling with blue-light responses, thereby linking environmental cues to floral scent emission [70].

In addition to transcriptional regulation, post-transcriptional and post-translational mechanisms also contribute to scent modulation. Protein phosphorylation, ubiquitination, and subcellular localization changes can influence the stability and catalytic activity of key enzymes such as TPSs, indicating that floral scent regulation extends beyond transcriptional control [91,115].

Overall, floral scent formation in Chinese *Cymbidium* orchids is controlled by a multilayered regulatory system integrating genetic determinants, metabolic flux, environmental signals, developmental cues, and complex signal transduction networks. This integrated regulation ensures both compositional diversity and spatiotemporal precision of volatile emission. Future research should adopt multi-omics strategies, including transcriptomics, metabolomics, proteomics, and epigenomics, to construct a comprehensive regulatory network of floral scent biosynthesis. Such integrative approaches will provide a theoretical foundation for precision breeding and metabolic engineering of fragrance traits in Chinese *Cymbidium* orchids.

## 5. Future Directions

The distinctive floral scents of Chinese *Cymbidium* orchids are important ornamental traits and chemical signals shaped by long-term adaptation to pollination ecology. Advances in volatile profiling, metabolomics, and molecular biology have improved the characterization of floral VOCs and their biosynthetic mechanisms [30,116]. Nevertheless, most studies have focused on volatile identification and a limited number of biosynthetic genes, whereas the regulatory networks, environmental responses, and ecological functions underlying scent formation remain poorly understood.

Future research should clarify the genetic and metabolic basis of scent diversity in Chinese *Cymbidium* orchids. Although terpene and MeJA biosynthesis has been extensively investigated, the pathways and regulatory networks governing phenylpropanoid/benzenoid and other fatty acid-derived volatiles remain largely unexplored. Characterization of individual structural genes, such as TPS and JMT genes, cannot fully explain the complexity of volatile accumulation. Greater attention should therefore be given to precursor availability, metabolic flux partitioning, transcriptional regulation, protein interactions, and post-translational modifications.

The relationships among scent formation, environmental responses, and ecological adaptation also require further investigation. Temperature, photoperiod, floral development, circadian rhythms, and phytohormones affect VOC emission, but their underlying regulatory mechanisms remain unclear. Direct evidence linking individual compounds or scent blends to pollinator preference is also limited. Integrating behavioral assays, electrophysiology, and field observations will be essential for determining how scent variation affects pollinator attraction, reproductive success, and adaptive diversification.

Reliable functional validation remains another major challenge. Many candidate genes have been characterized mainly through heterologous expression, which does not fully reflect their functions in native *Cymbidium* backgrounds. Efficient transformation, gene-editing, and transient-expression systems are therefore needed to validate gene function and accelerate the use of scent-related genetic resources [22,24].

Overall, future studies should integrate genomics, transcriptomics, metabolomics, functional genetics, and chemical ecology. Such multidisciplinary approaches will clarify the molecular and evolutionary basis of floral scent diversity and support germplasm conservation and the breeding of fragrant *Cymbidium* cultivars.

## 6. Conclusions

The floral scents of Chinese *Cymbidium* orchids consist mainly of terpenoids and fatty acid-derived compounds, together with smaller proportions of phenylpropanoid/benzenoid volatiles. Terpenes and MeJA-related compounds contribute substantially to species-specific scent profiles. Differences in VOC composition among species reflect distinct metabolic strategies and may be associated with pollination ecology and reproductive evolution.

Research has progressed from descriptive volatile profiling toward the characterization of biosynthetic and regulatory mechanisms. The MVA and MEP pathways, together with *TPS* genes, drive terpene biosynthesis and diversification. The LOX–AOS–AOC–OPR–JMT pathway and its associated transcription factors regulate MeJA biosynthesis. Tissue-specific, developmental, and diel emission patterns further demonstrate that scent formation is coordinated by genetic, developmental, and environmental signals.

However, the biosynthesis and regulation of phenylpropanoid/benzenoid and other minor volatiles remain poorly characterized. The contributions of metabolic flux, protein interactions, and post-translational regulation are also largely unknown. Moreover, limited functional validation in native *Cymbidium* backgrounds and insufficient evidence linking scent composition to pollinator behavior restrict mechanistic and ecological interpretation (Figure 5).

Integrating multi-omics analyses, functional genetics, and chemical ecology will advance understanding of scent formation, diversification, and pollinator interactions in Chinese *Cymbidium* orchids. These insights will also provide a theoretical basis for orchid conservation and the molecular improvement of fragrant cultivars.

## Figures and Tables

**Figure 1:**
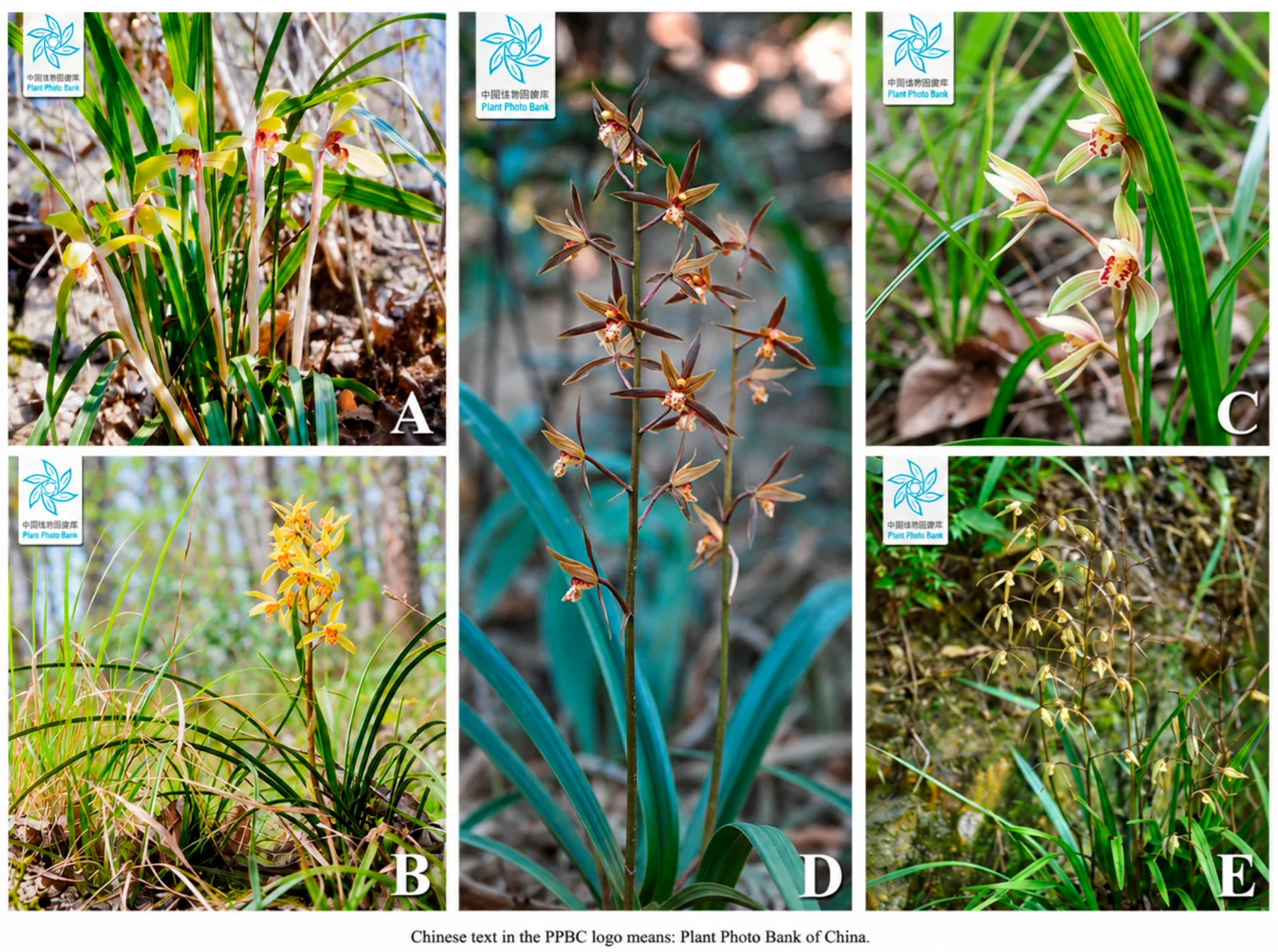
Morphological diversity of five representative Chinese *Cymbidium* orchids. (**A**) *Cymbidium goeringii*; (**B**) *Cymbidium faberi*; (**C**) *Cymbidium ensifolium*; (**D**) *Cymbidium sinense*; and (**E**) *Cymbidium kanran.* Images were obtained from the Plant Photo Bank of China (PPBC, https://ppbc.iplant.cn/). The photographs were taken in different natural distribution regions of China, including Henan, Hubei, Fujian, and Hunan Provinces. All plant materials were taxonomically identified and verified by qualified botanists to ensure accurate species identification (the morphological characteristics, flowering phenology, and fragrance traits of these Chinese orchids are placed in Supplementary Table S1).

**Figure 2:**
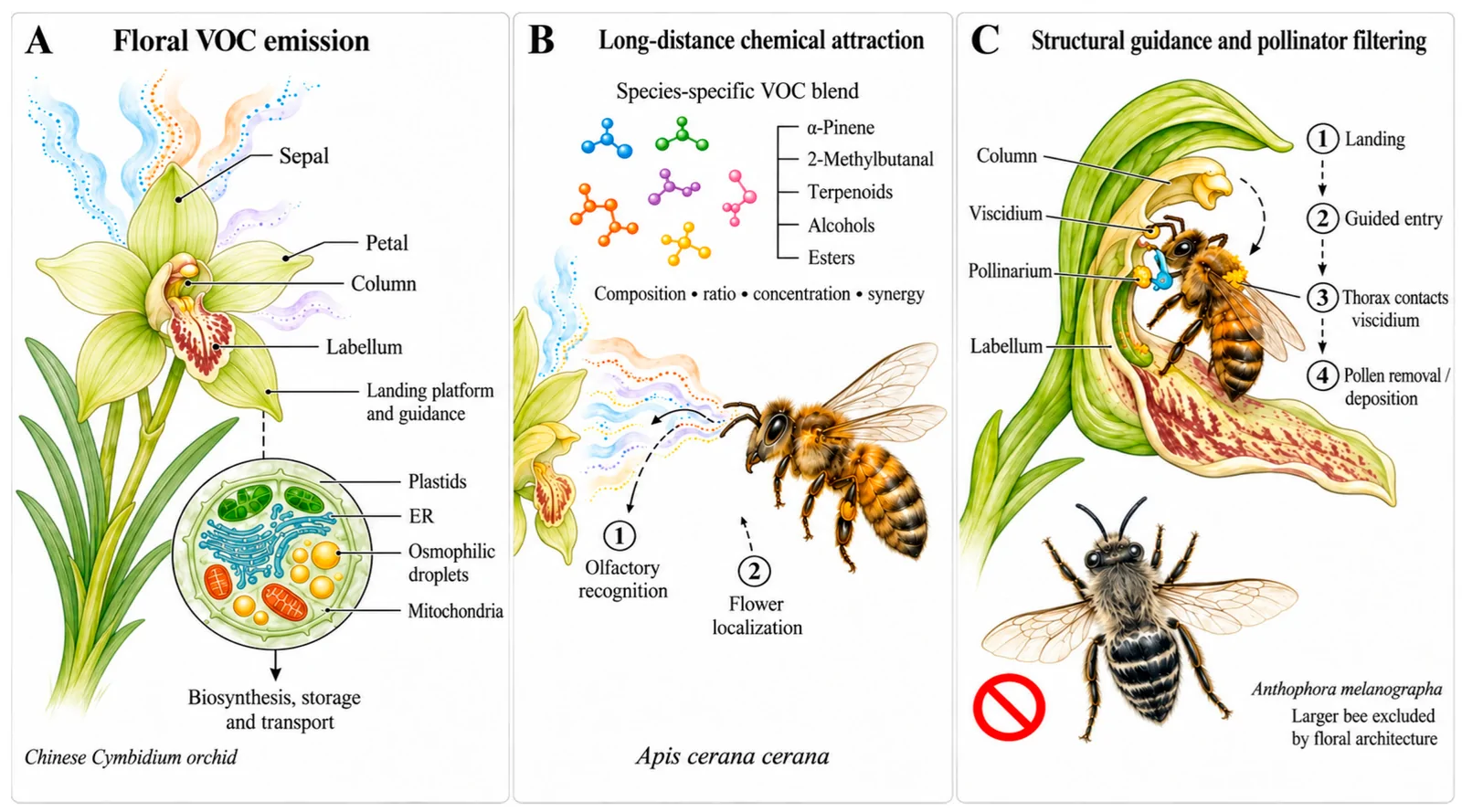
Integration of Floral Volatiles and Floral Architecture in Pollinator Attraction and Selection of Chinese *Cymbidium* Orchids.

**Figure 3:**
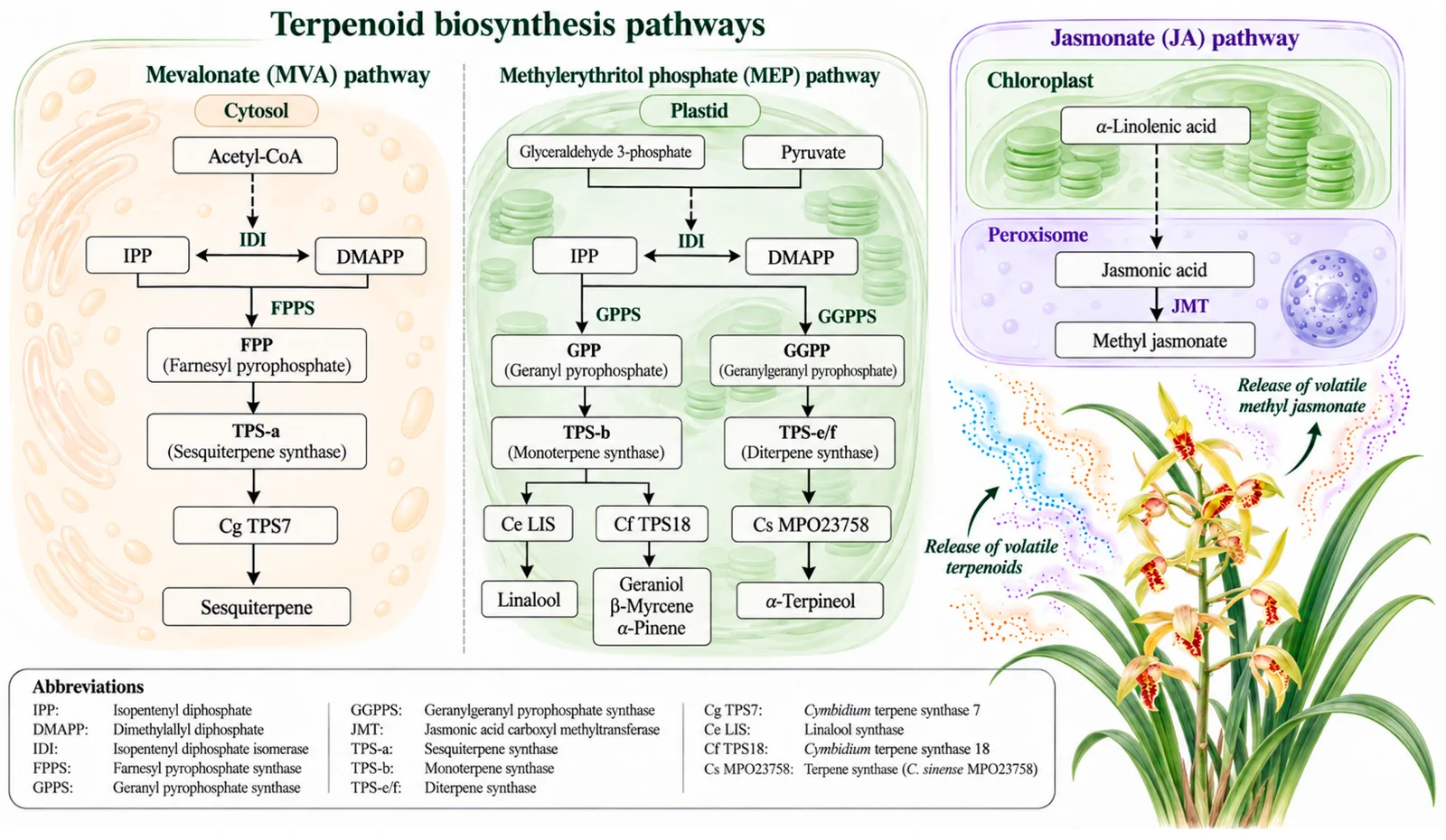
Simplified biosynthetic pathways of terpenoid and methyl jasmonate floral volatiles in Chinese *Cymbidium* orchids.

**Figure 4:**
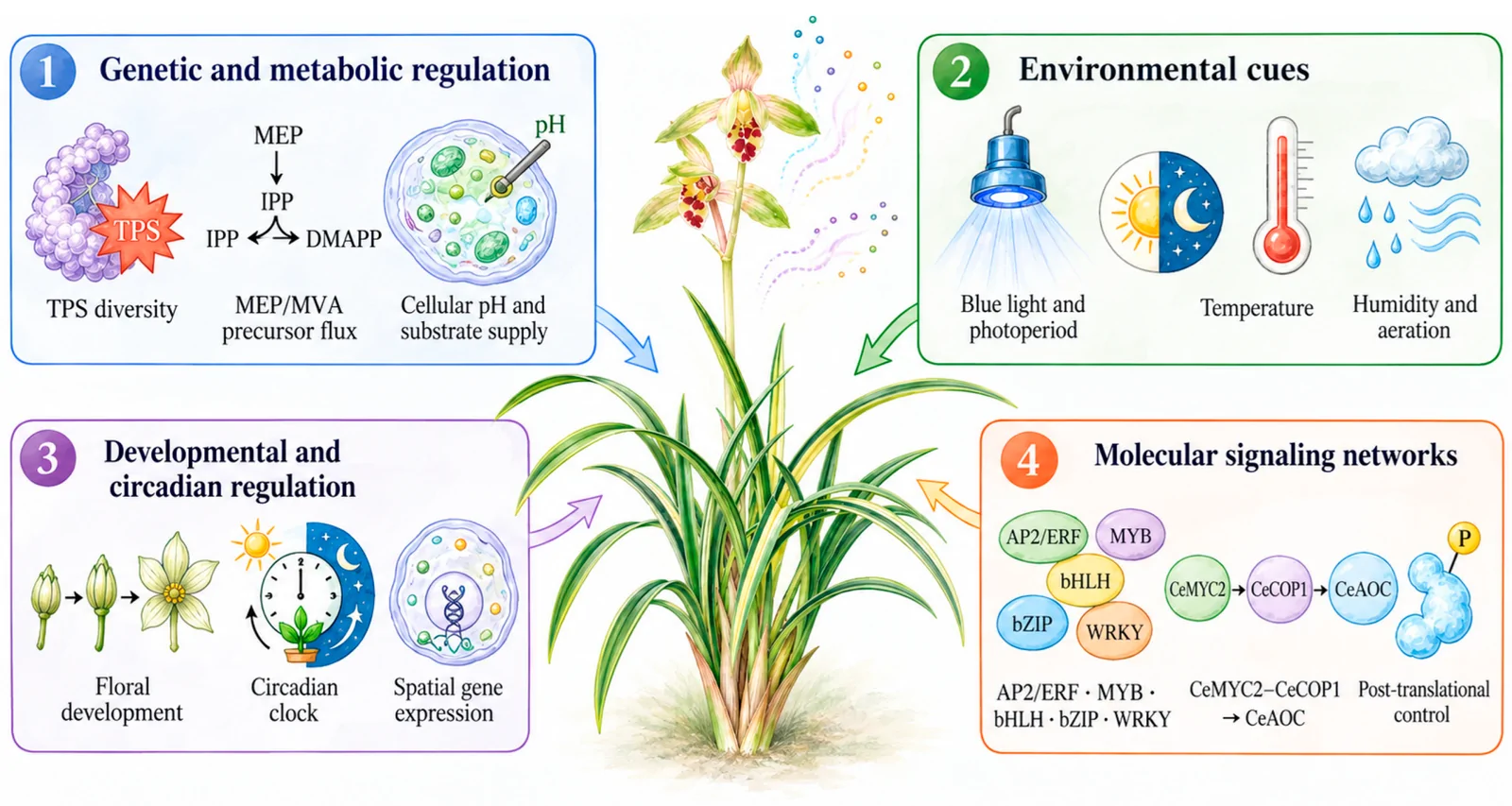
Integrated regulatory framework of floral scent emission in Chinese *Cymbidium* orchids. Integrated model illustrating the major regulatory factors affecting floral scent emission in Chinese *Cymbidium* orchids. The interaction among these factors collectively determines the biosynthesis, accumulation, and release of floral VOCs.

**Figure 5:**
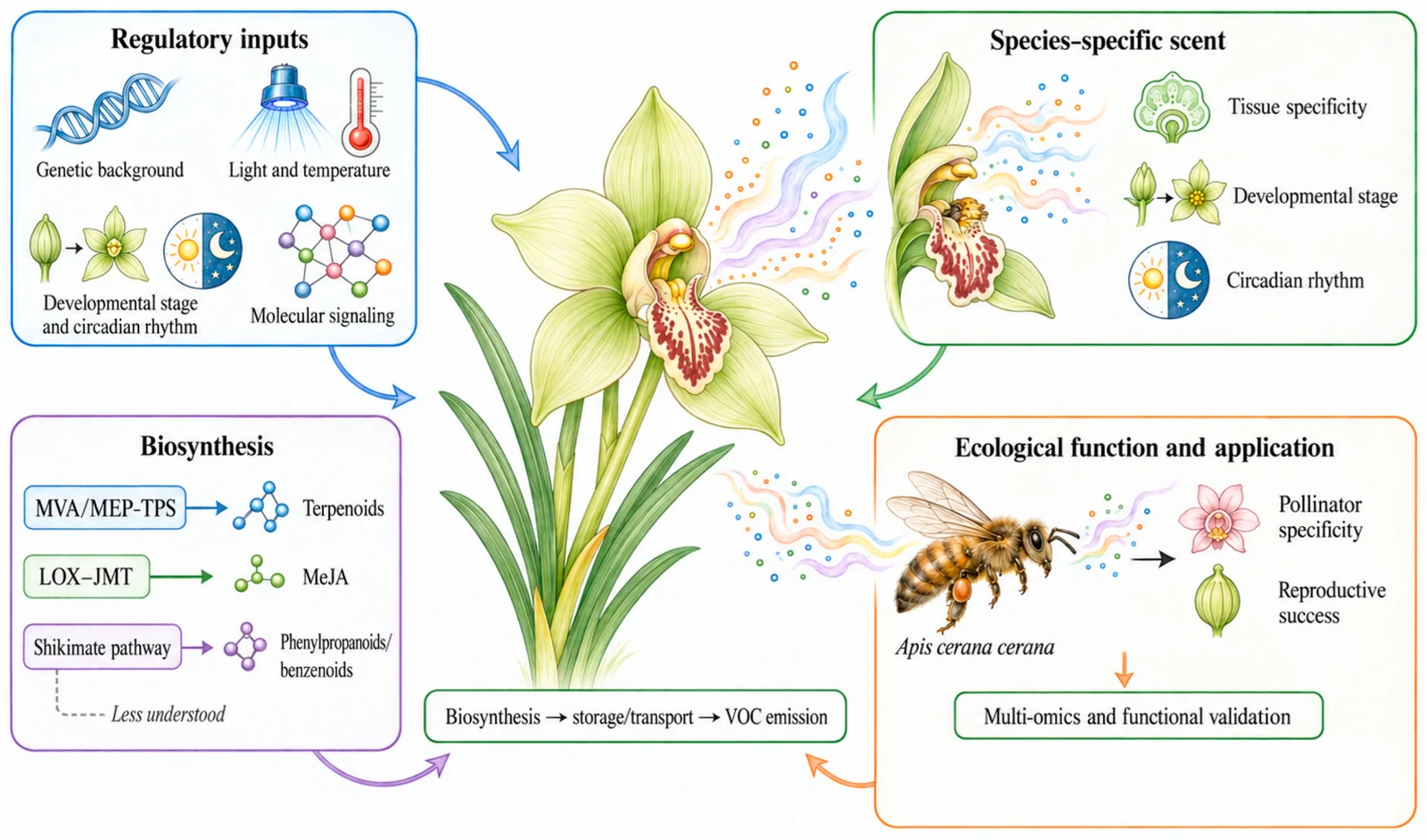
Future perspectives and integrated research framework for understanding floral scent formation and ecological functions in Chinese *Cymbidium* orchids.

**Table 1:** Summary of studies on the main volatile components in the flowers of some Chinese *Cymbidium* species. The data in the table are compiled from published literature. The volatile compounds listed represent the major or relatively abundant floral scent components reported in the respective studies, and do not constitute the complete volatile profiles of the species. Due to differences in sampling time, source of plant material, cultivation environment, and volatile collection/detection conditions (e.g., extraction temperature, extraction duration, and fiber type), some discrepancies may exist among results from different studies. N/A indicates that the original publication did not provide the relevant information.

Species	Region	Detection Method, Extraction Temperature & Time	Main Volatile Compounds	References
*Cymbidium goeringii*	Japan	Tenax-GC/MS, 180 °C, 40 min	nerolidol, methyl jasmonate, pentane, limonene, caryophyllene	(Omata et al., 1990) [41]
	Henan Province, China	HS-SPME-GC/MS, 50 °C, 60 min	α-farnesene, palmitic acid, methoxyphenyloxime, cadinene, cedrene	(Peng, 2009) [42]
	Beijing Municipality, China	HS-SPME-GC/MS, 20 °C, 60 min	nerolidol, santalol, 1,2,3,4-tetrahydro-1,6,8-trimethyl-naphthalene	(Yang, 2011) [43]
	Zhejiang Province, China	HS-SPME-GC/MS, 25 °C, 90 min	α-selinene, dodecane, n-tetradecane, thujopsene	(Wei et al., 2013) [44]
	Guizhou Province, China	HS-SPME-GC/MS, 65 °C, 30 min	β-farnesene, α-farnesene, nerolidol, 1,9-decadiyne	(Fang et al., 2013) [45]
	Qinling Mountains Region, China	HS-SPME-GC/MS, 65 °C, 35 min	3-ethyl-2-methyl-1,3-hexadiene, α-bergamotene, 3-methoxy-5-methylphenol, 2-octen-1-ol, farnesol, nonanal	(Chen et al., 2016) [46]
	Taiwan Region, China	HS-SPME-GC/MS, 60 °C, 5 min	farnesol, epi-methyl jasmonate, β-farnesene, nerolidol	(Ramya et al., 2019) [47]
	Jiangxi Province, Chinai	HS-SPME-GC/MS, 60 °C, 40 min	nerolidol, farnesol, α-bergamotene, benzyl alcohol, hexanal, heptanal, 4-methylcyclohexanol, nonanal, methyl heptenone	(Zhou, 2020) [48,49]
	Guizhou Province, China	HS-SPME-GC/MS, 80 °C, 30 min	nerolidol, (2-methyl-2-propen-1-Yl) succinic anhydride, guaiazulene	(Jin et al., 2021) [50]
	Sichuan Province, China	HS-SPME-GC/MS, 40 °C, 15 min	α-caryophyllene, p-methylanisole, benzyl alcohol, phenylethyl alcohol	(Wang et al., 2024) [51]
*Cymbidium faberi*	Japan	Tenax-GC/MS, 180 °C, 4 min	epi-methyl jasmonate, methyl jasmonate, farnesol, β-farnesene, limonene	(Omata et al., 1990) [41]
	Henan Province, China	HS-SPME-GC/MS, 50 °C, 60 min	methyl jasmonate, epi-methyl jasmonate, farnesol, β-farnesene, ocimene	(Peng, 2009) [42]
	Beijing Municipality, China	HS-SPME-GC/MS, 20 °C, 60 min	2,5-dimethyl-1,5-hexadiene-3,4-diol, nerolidol, methyl jasmonate, ocimene, farnesol	(Yang, 2011) [43]
	Zhejiang Province, China	HS-SPME-GC/MS, 25 °C, 90 min	eucalyptol, n-tetradecane, methyl jasmonate, α-thujene, farnesol	(Wei et al., 2013) [44]
	Guizhou Province, China	HS-SPME-GC/MS, 65 °C, 30 min	α-caryophyllene, β-pinene, nonanal, valencene, thymol methyl ether	(Fang et al., 2013) [52]
	Qinling Mountains Region, China	HS-SPME-GC/MS, 65 °C, 35 min	nerolidol, methyl jasmonate, docosahexaenoic acid, calamenene, α-ocimene, α-farnesene	(Chen et al., 2016) [46]
	Jiangxi Province, China	HS-SPME-GC/MS, 60 °C, 40 min	2-ethylbutanal, β-ocimene, p-mentha-1,3,8-triene, 2,5-dimethyl-1,5-hexadiene-3,4-diol	(Zhou, 2020) [48,49]
	Jiangsu Province, China	HS-SPME-GC/MS, 45 °C, 40 min	farnesol, α-guaiene, methyl jasmonate, β-caryophyllene, linalyl formate, dihydro-α-ionone, calamenene, viridiflorene, octyl isobutyrate, α-selinene, heliotropin, α-longipinene, sabinene, tetrahydrogeraniol, alloaromadendrene, geraniol	(Yuan et al., 2019) [53]
	Zhejiang Province, China	HS-SPME-GC/MS, 80 °C, 40 min	methyl ethyl acetaldehyde, 2-pentylfuran, butyl acetate	(Huang et al., 2026) [54]
*Cymbidium ensifolium*	Zhejiang Province, China	HS-SPME-GC/MS, 60 °C, 40 min	methyl jasmonate, epi-methyl jasmonate, 12-[4.4.3.0(1,6)]oxatricyclotridecane-3,11-dione, Z,Z,Z-1,4,6,9-nonadecatetraene	(Peng, 2009) [42]
	Beijing Municipality, China	HS-SPME-GC/MS, 20 °C, 60 min	epi-methyl jasmonate, farnesol, 4,7,10,13,16,19-docosahexaenoic acid methyl ester, methyl jasmonate, butanedioic acid, methyl-bis(1-methylpropyl) ester	(Yang, 2011) [43]
	Jiangxi Province, China	HS-SPME-GC/MS, 45 °C, 40 min	methyl jasmonate, epi-methyl jasmonate, docosahexaenoic acid, 2,2,4-trimethylpentanediol isobutyrate	(Zhou, 2020) [48,49]
	Guangdong Province, China	HS-SPME-GC/MS, 25 °C, 60 min	methyl jasmonate, epi-methyl jasmonate, β-farnesene, β-bisabolene, farnesol	(Gao et al., 2024) [55]
*Cymbidium sinense*	Zhejiang Province, China	HS-SPME-GC/MS, 25 °C, 90 min	methyl isoeugenol, methyl 6-oxoheptanoate, isoelemicin, benzyl alcohol	(Wei et al., 2013) [44]
	Guangdong Province, China	HS-SPME-GC/MS, N/A	farnesol, n-tetracosanol, dihydro-α-ionone, β-ionone, pentacosane, heptacosane, α-ionene	(Li et al., 2017) [56]
	Jiangxi Province, China	HS-SPME-GC/MS, 60 °C, 40 min	β-ionone, dihydro-α-ionone, farnesol	(Zhou, 2020) [48]
	Guangdong Province, China	HS-SPME-GC/MS, N/A	linalool, α-terpineol, β-cyclocitral, (E,E)-2,4-decadienal, β-farnesene, α-farnesene, β-bisabolene, farnesol, farnesal	(Yang et al., 2021) [57]
	Guangxi Province, China	HS-SPME-GC/MS, 40 °C, 30 min	pentanal, hexanal, E-2-pentenal, E-2-hexenal, cyclopentanecarboxaldehyde, E-2-heptenal, 1-penten-3-Ol, E-2-penten-1-Ol	(Ding et al., 2023) [58]
	Sichuan Province, China	HS-SPME-GC/MS, 40 °C, 15 min	α-pinene, limonene, ethyl hexanoate, methyl heptenone	(Wang et al., 2024) [51]
	Zhejiang Province, China	HS-SPME-GC/MS, 80 °C, 40 min	methyl ethyl acetaldehyde, 2-pentylfuran, 2-nonenal	(Huang et al., 2026) [54]
*Cymbidium kanran*	Beijing Municipality, China	HS-SPME-GC/MS, 50 °C, 60 min	methyl jasmonate, epi-methyl jasmonate, methyl 2,5-octadecadiynoate, farnesyl acetate	(Peng, 2009) [42]
	Beijing Municipality, China	HS-SPME-GC/MS, 20 °C, 60 min	epi-methyl jasmonate, methyl jasmonate, docosahexaenoic acid methyl ester, farnesol	(Yang, 2011) [43]
	Jiangxi Province, China	HS-SPME-GC/MS, 60 °C, 40 min	α-bergamotene, 2-pentylfuran, nonanal, 3-ethyl-2-methyl-1,3-hexadiene, (E)-2-octenal	(Zhou Yalian, 2020) [48]
	Jiangxi Province, China	HS-SPME-GC/MS, N/A	pentanal, hexanal, 2-pentylfuran, N-(1-oxopentyl)-L-alanine methyl ester, methyl isobutyl ketone, 1,1-dimethylcyclopropane, 1,4-cyclooctadiene	(Luo et al., 2024) [59]

**Table 2:** Major floral volatile compounds and characteristic volatile composition of five Chinese *Cymbidium* orchids. This table was compiled based on published floral scent analyses of *Cymbidium goeringii*, *C. faberi*, *C. ensifolium*, *C. sinense*, and *C. kanran*. According to their chemical structures, volatile compounds were classified into four major groups: terpenoids (monoterpenes mainly synthesized through the methylerythritol phosphate (MEP) pathway and sesquiterpenes mainly derived from the mevalonate (MVA) pathway), carotenoid-derived volatiles (primarily produced through the carotenoid cleavage dioxygenase (CCD) pathway), phenylpropanoid/benzenoid compounds (mainly derived from the shikimate pathway), and fatty acid-derived compounds (including jasmonates mainly synthesized through the lipoxygenase (LOX) pathway, as well as other esters, aldehydes, and hydrocarbons mainly associated with fatty acid synthase (FAS), fatty acid elongase (FAE), and cuticular wax alkane biosynthesis pathways). Only representative compounds with high detection frequencies or relatively high abundances within each chemical category are presented, together with their structural formulas.

Chemical Class	Monoterpenes	Sesquiterpenes	Apocarotenoids	Phenylpropanoids /Benzenoids	Jasmonates	Fatty Acid-Derived Aldehydes	Other Fatty Acid Derivatives	Dominant Compounds
Representative Compounds	linalool, α-terpineol, limonene, ocimene, pinene, etc.	nerolidol, farnesol, farnesene, caryophyllene, etc.	β-ionone, dihydro-α-ionone, etc.	phenylethanol, benzyl alcohol, methyl isoeugenol, etc.	methyl jasmonate, epi-methyl jasmonate	hexanal, nonanal, heptanal, etc.	long-chain esters, long-chain alkanes, etc.	—
*C. goeringii*	trace	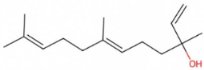 nerolidol 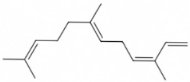 α-farnesene 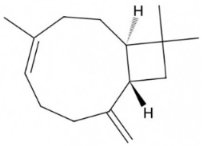 caryophyllene	—	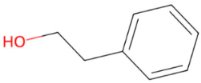 phenylethanol 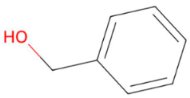 benzyl alcohol	trace	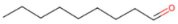 nonanal 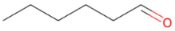 hexanal	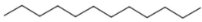 dodecane 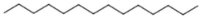 tetradecane	nerolidol
*C. faberi*	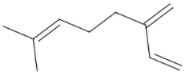 ocimene 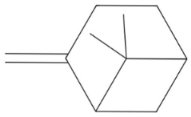 β-pinene (trace)	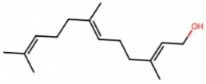 farnesol 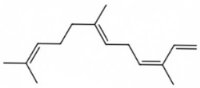 α-farnesene 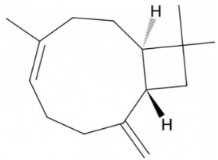 caryophyllene	trace	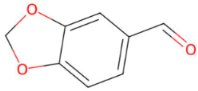 piperonal	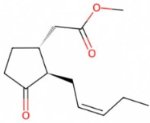 methyl jasmonate 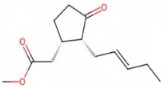 epi-methyl jasmonate	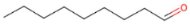 nonanal	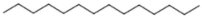 tetradecane	farnesol, methyl jasmonate
*C. ensifolium*	trace	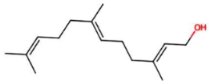 farnesol 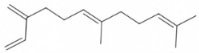 β-farnesene	—	trace	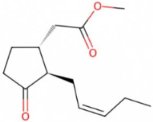 methyl jasmonate 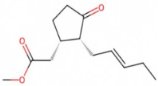 epi-methyl jasmonate	trace	long-chain esters	methyl jasmonate
*C. sinense*	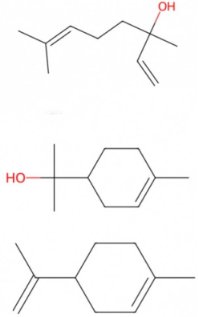 limonene (trace)	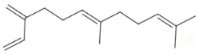 β-farnesene	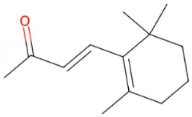 β-ionone 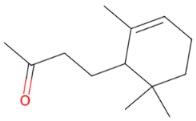 dihydro-α-ionone	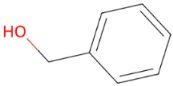 benzyl alcohol 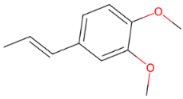 methyl isoeugenol (trace)	trace	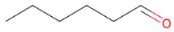 hexanal 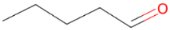 pentanal	long-chain alkanes	linalool, β-ionone
*C. kanran*	trace	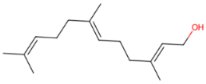 farnesol 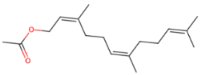 farnesyl acetate	—	trace	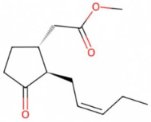 methyl jasmonate 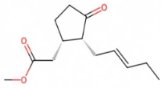 epi-methyl jasmonate	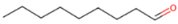 nonanal	long-chain esters	methyl jasmonate

## Data Availability

No new data were created or analyzed in this study. Data sharing is not applicable to this article.

## Supplementary Materials

The following supporting information can be downloaded at: https://www.mdpi.com/article/10.3390/plants15192907/s1, Figure S1: floral organs of *Cymbidium faberi*., including dorsal sepal, lateral sepals, petals, column, and labellum (lip); Table S1: Morphological characteristics, flowering phenology, and fragrance traits of major Chinese *Cymbidium* orchids in China.
