# Supplementary material for: Floral Volatile Organic Compounds of Chinese Cymbidium Orchids: From Biosynthesis and Function to Genetic Regulation
Source: Plants (Basel). 2026 Sep 23;15(19):2907. doi: 10.3390/plants15192907 (PMC13666902; doi:10.3390/plants15192907)
Supplement: Supplementary file 1 [file plants-15-02907-s001.zip › Supplementary Figure S1.pdf]

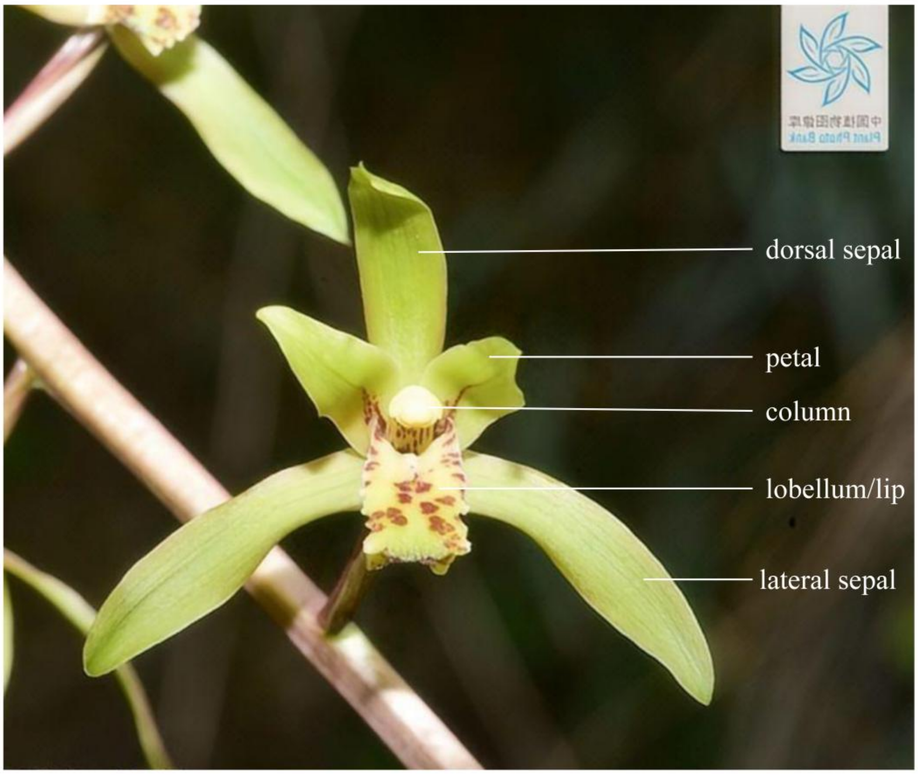

Supplementary Figure S1. floral organs of *Cymbidium faberi*., including dorsal sepal, lateral sepals, petals, column, and labellum (lip).Images were obtained from the Plant Photo Bank of China (PPBC, https://ppbc.iplant.cn/). The photographs were taken from natural populations of *C. faberi* in Shennongjia, Hubei Province, China.Specimen were identified and authenticated by professional taxonomists.
