# Supplementary material for: Floral Volatile Organic Compounds of Chinese Cymbidium Orchids: From Biosynthesis and Function to Genetic Regulation
Source: Plants (Basel). 2026 Sep 23;15(19):2907. doi: 10.3390/plants15192907 (PMC13666902; doi:10.3390/plants15192907)
Supplement: Supplementary file 1 [file plants-15-02907-s001.zip › Supplementary Table S1.pdf]

Supplementary Table S1. Morphological characteristics, flowering phenology, and fragrance traits of major Chinese *Cymbidium* orchids in China.Flowering periods represent the typical phenology of Chinese *Cymbidium* orchids under natural conditions in China and may vary depending on geographic distribution, altitude, climate conditions, and cultivation environment.

Characteristics	*Cymbidium goeringii*	*Cymbidium faberi*	*Cymbidium ensifolium*	*Cymbidium sinense*	*Cymbidium kanran*
**Plant habit**	Small-sized plants. Leaves usually clustered, narrow and elongated.	Medium-to-large plants with robust growth.	Medium-sized plants with relatively compact morphology.	Large plants with broad leaves and vigorous growth.	Medium-sized plants with upright growth habit.
**Leaf characteristics**	Leaves 4 – 6 per shoot, narrowly linear, approximately 20 – 40 (up to 100) cm long and 0.6 – 1.1 cm wide; margins slightly serrated.	Leaves 5 – 7 per shoot, 25 – 80 cm long and approximately 1 cm wide; erect, thick and rigid, with prominent midrib and slightly serrated margins.	Leaves 2 – 6 per shoot, 30 – 50 cm long and 1.2 – 1.7 cm wide; glossy and slightly curved.	Leaves 4 – 5 per shoot, sword-shaped, 60 – 80 cm long and 2.7 – 4.2 cm wide; leathery, glossy, and slightly spreading.	Leaves 3 – 7 per shoot, erect, 35 – 70 cm long and 1 – 1.7 (up to 2.5) cm wide; leathery with slightly glossy surface.
**Inflorescence and flower traits**	Usually single-flowered inflorescences, occasionally with two flowers. Flower stalks shorter than leaves. Flowers 4 – 5 cm in diameter, yellow-green to greenish-white, fragrant.	Erect inflorescences, 30 – 80 cm long, bearing 5 – 12 (occasionally 18) flowers. Flowers pale yellow-green, 5 – 6 cm in diameter, strongly fragrant.	Erect inflorescences, approximately 25 – 35 cm long, generally shorter than leaves, with 5 – 9 (up to 13) flowers. Flowers pale yellow-green, 4 – 6 cm in diameter, fragrant.	Erect inflorescences usually exceeding leaves, bearing 7 – 17 flowers. Flowers pale yellow-brown with purple veins; sepals narrow and petals spreading. Fragrant.	Erect inflorescences, generally equal to or longer than leaves, bearing approximately 10 flowers. Flowers yellow-green with purple veins; petals narrow and spreading. Fragrant.
**Flowering period**	February – March.	March – April.	July – October (occasionally flowering twice annually from summer to autumn).	September – March of the following year.	Varies among geographical populations; generally flowering from July, with peak flowering from November to January.
